# Linking phylogeny to abundant ribotypes of community fingerprints: an exercise on the phylotypic responses to plant species, fertilisation and *Lolium perenne* ingression

**DOI:** 10.1186/2193-1801-2-522

**Published:** 2013-10-09

**Authors:** Ann-Kathrin Liliensiek, Dwipendra Thakuria, Nicholas Clipson, Narayan C Talukdar

**Affiliations:** School of Biology and Environmental Sciences, University College Dublin, Belfield, Dublin 4 Ireland; Department of Aquatic Chemistry, Federal Institute of Hydrology, Am Mainzer Tor 1, Koblenz, 56068 Germany; Department of Biotechnology, Institute of Bioresources and Sustainable Development, Microbial Resources Division, GOI, Imphal, Manipur India

**Keywords:** ARISA, Grassland, Land use, Phylogeny, Plant species, Soil type

## Abstract

The present study explores the potential of directly linking phylogenetic identities obtained by cloning and sequencing of ITS sequences to dominant ribotypes of molecular community fingerprints to give further insight into dominant members of the communities in three Irish grassland soils. The ten most abundant bacterial ribotypes of untreated bare soils of three grassland microcosms were chosen to represent the “baseline community” of the respective soil. Identities on phylum and order level were assigned to these ribotypes on a weighted basis, by matching sequence homologies of cloned ITS sequences with ribotypes of the same fragment lengths ±5 bp. Results showed that ribotypes were represented by the phyla Acidobacteria, Actinobacteria, Proteobacteria, and Firmicutes and the distribution of the ribotype and phylotype communities was shown to be highly site-specific. Furthermore the response of dominant bacterial phylotypes to plant species composition, fertilisation and *Lolium perenne* ingression was investigated within a larger microcosm study (Microb Ecol 63:509–521).

**Electronic supplementary material:**

The online version of this article (doi:10.1186/2193-1801-2-522) contains supplementary material, which is available to authorized users.

## Main text

Microbial communities are likely to be influenced by a wide range of factors, including environmental and anthropogenic factors. In order to examine those influences on the community structure, molecular fingerprinting techniques, such as terminal restriction fragment length polymorphism (TRFLP) or automated ribosomal intergenic spacer analysis (ARISA), are commonly employed (Thies 2007). While these automated fingerprinting techniques provide insight into changes of community structure at high resolutions, they give little or no information on the community members involved in the change. To tackle the question, whether or not changes in ribotype communities actually reflect significant changes on a phylogenetic or functional level, some studies employ amplification of functional or group-specific genes prior to fingerprinting (Briones et al. 2002; O’Callaghan et al. 2008), while other studies complement fingerprinting with cloning and sequencing analysis to identify species present in samples (Dickie & Fitzjohn 2007; Shi and Bending 2007; Shi et al. 2011). This study explored the latter approach and its potential of directly linking cloning and sequencing data to ARISA-derived ribotypes to provide further insight into community changes induced by agricultural practices in three different Irish grassland soils (Liliensiek et al. 2012; for soil properties see Additional file 1: Table S1), including a near-neutral grassland (hereafter called Burren soil), a mesotrophic grassland (Ardgillan soil), and an acidic upland grassland (Wicklow soil). The phylogenetic identities of ARISA-derived dominant members were projected onto the fingerprinting data of a larger microcosm study (Liliensiek et al. 2012; Additional file 2: Table S2) to investigate the response of dominant bacterial phylotypes to plant species composition, fertilisation and *Lolium perenne* ingression.

In order to assign phylogenetic identities to the 10 most abundant bacterial ARISA ribotypes (hereafter referred to as baseline ribotypes) of each untreated bare soil type (indicated by shaded cells in Additional file 2: Table S2), PCR products (primers ITSF/ITSReub, Cardinale et al. 2004) of DNA extracted from microcosms of each of the untreated bare soil types (Liliensiek et al. 2012) were randomly cloned (pGEM-T easy, Promega), sequenced (primers SP6/T7, Macrogen Inc.) and matched to homologies in the NCBI database using BLAST (Altschul et al. 1997; see Additional file 3: Methods). Clones were then assigned to the baseline ribotypes based on corresponding sequence lengths in order to confer phylogenetic identities to ribotypes. Sequences targeting the ITS region have previously been applied to phylogenetic analyses (Ranjard et al. 2000), but contain only short stretches of the 16S and 23S genes. Furthermore, sequences in the database are based on culturable organisms with potentially low sequence homologies to environmental 16S genes (van Wintzingerode et al. 1997). Thus not all ribotypes could be exactly matched with a phylogenetically identified clone. Tolerating a mismatch in length of ±5 bp between ribotype number and cloned sequence length, however, about 67% of ribotypes present in the baseline-communities could be assigned down to phylum level (Additional file 4: Table S3). Given a certain degree of inaccuracy during fragment analysis as well as sequencing, such a variation was deemed possible to occur. In order to limit the resulting bias, matches between ribotypes and their respective clones were assigned on a weighted basis. Weighted abundances of ribotypes associated to the same phylum were then averaged to give an estimation of the phylogenetic distribution (see Additional file 3: Methods; Table 1).

**Table 1:** **Means and variations of relative abundances (rfu) of top 10 ribotypes and associated phyla as well as ANOVA F-values for their distribution across bare untreated soils**

Ribotype	NCBI matches	Top 10 of site	Relative abundances (rfu)			ANOVA
			Burren	Ardgillan	Wicklow	
**225**	ni	W,A,B	1480±130 ^*a*^	2704±259 ^*a*^	2265±432 ^*a*^	4.26 ^ns^
**241**	ni	W	0±0 ^*c*^	538±83 ^*b*^	1796±212 ^*a*^	49.08^***^
**282**	An, αP, γP	B	2982±272 ^*a*^	488±116 ^*b*^	0±0 ^*b*^	87.99^***^
**301**	An, F	A	872±124 ^*a*^	1444±165 ^*a*^	803±341 ^*a*^	2.34 ^ns^
**302**	An, F	A	0±0 ^*b*^	1499±82 ^*a*^	0±0 ^*b*^	333.49^***^
**324**	ni	B	1323±102 ^*a*^	422±38 ^*b*^	0±0 ^*c*^	115.58^***^
**352**	ni	W	0±0 ^*b*^	0±0 ^*b*^	1643±206 ^*a*^	63.84^***^
**390**	An, F	W	0±0 ^*b*^	0±0 ^*b*^	4387±308 ^*a*^	202.24^***^
**391**	An, F	W	0±0 ^*b*^	0±0 ^*b*^	1634±204 ^*a*^	64.24^***^
**398**	An, F	W	0±0 ^*c*^	705±16 ^*b*^	1538±328 ^*a*^	16.49^**^
**400**	An	A	647±48 ^*b*^	3704±106 ^*a*^	1158±237 ^*b*^	115.20^***^
**409**	An	W	0±0 ^*c*^	853±108 ^*b*^	1667±29 ^*a*^	168.33^***^
**417**	An	B	1938±63 ^*a*^	0±0 ^*c*^	214±65 ^*b*^	411.27^***^
**502**	γP	B	1427±35 ^*a*^	0±0 ^*b*^	0±0 ^*b*^	1702.14^***^
**612**	ni	B	4103±85 ^*a*^	0±0 ^*b*^	0±0 ^*b*^	2330.72^***^
**831**	F	A	885±96 ^*b*^	2327±132 ^*a*^	0±0 ^*c*^	154.64^***^
**835**	F	A	1190±83 ^*b*^	2002±175 ^*a*^	0±0 ^*c*^	80.81^***^
**843**	F	A	0±0 ^*b*^	1183±76 ^*a*^	0±0 ^*b*^	244.56^***^
**846**	ni	A	0±0 ^*b*^	3296±153 ^*a*^	0±0 ^*b*^	463.38^***^
**850**	F	B	4376±476 ^*a*^	766±122 ^*b*^	0±0 ^*b*^	67.94^***^
**853**	F	B	3241±212 ^*a*^	0±0 ^*b*^	0±0 ^*b*^	234.04^***^
**890**	ni	A,B	2742±125 ^*a*^	2021±512 ^*a*^	0±0 ^*b*^	21.82^**^
**895**	F	B	1411±121 ^*a*^	0±0 ^*b*^	0±0 ^*b*^	135.38^***^
**904**	F	A	953±13 ^*b*^	1167±95 ^*a*^	0±0 ^*c*^	126.18^***^
**917**	ni	W	509±121 ^*b*^	0±0 ^*c*^	2216±118 ^*a*^	141.19^***^
**920**	ni	W	0±0 ^*b*^	0±0 ^*b*^	9694±612 ^*a*^	250.79^***^
**953**	Ad	W	0±0 ^*b*^	0±0 ^*b*^	1555±258 ^*a*^	36.33^***^
**Phylotype**						
**Acidobacteria**		W,A,B	0±0 ^*b*^	0±0 ^*b*^	13998±2322 ^*a*^	36.33^***^
**Actinobacteria**		W	17202±678 ^*a*^	11368±240 ^*b*^	9051±500 ^*c*^	69.05^***^
**α-Proteobacteria**		B	16234±1365 ^*a*^	0±0 ^*b*^	0±0 ^*b*^	141.36^***^
**γ-Proteobacteria**		B	17702±1239 ^*a*^	0±0 ^*b*^	0±0 ^*b*^	204.12^***^
**Firmicutes**		W,A,B	13325±384 ^*b*^	8887±191 ^*c*^	17157±890 ^*a*^	52.66^***^

Means and variations of the relative abundances in relative fluorescent units (rfu) of individual ribotypes and associated phyla (see Additional file 3: Methods) are presented for each site. Column three indicates the membership in the respective baseline community {Burren (B), Ardgillan (A), Wicklow (W)}. Superscript letters indicate the group differences between relative abundances across sites. The significance of distribution differences between sites are presented in the last column by F-values and their significance level (p = 0.05 ≥ * > 0.01 ≥ ** > 0.001 ≥ ***).

*Abbreviations*: ni – not identified, An – Actinobacteria, Ad – Acidobacteria, F – Firmicutes, αP – α-Proteobacteria, γP – γ-Proteobacteria.

Ribotypes present in the baseline-communities were represented by the phyla Acidobacteria, Actinobacteria, α-Proteobacteria, γ-Proteobacteria and Firmicutes, all of which have frequently been found in grassland soil (van Elsas et al. 2002). The distribution of these phyla among the top 10 ribotypes was however, significantly differed between soil types (Table 1), as shown by ANOVA (Additional file 3: Methods). Actinobacteria and Firmicutes were the most common and abundant phyla considering all soils combined. These phyla generally include the genera *Streptomyces*, *Clostridium* and *Bacillus*, which have been reported to exhibit the highest copy numbers (10–12 copies) of the RNA-operon (Tourova 2003). Thus, unless ribotypes can be identified down to genera-level, it remains unclear if individuals represented by abundant ribotypes are indeed highly abundant, or just appear as such due to a higher RNA-operon copy number.

While Actinobacteria and Firmicutes were represented by baseline-ribotypes from all three soils, Proteobacteria were solely present in the baseline-community of the Burren soil which is in agreement with their preference of a more alkaline pH (Kumar and Nicholas 1983). Acidobacteria, which are moderately acidophilic (Hugenholtz et al. 1998), on the other hand were found in the baseline-community of the Wicklow soil only.

Despite their presence in the baseline-communities of all three soils, the distribution of Actinobacteria and Firmicutes was significantly affected by soil type, whereby all soil properties (sand, silt, clay, pH and nitrogen and carbon content) contributed significantly to this effect for Firmicutes, but not for Actinobacteria, where pH and its interaction with clay were the most significantly contributing factors. The reputed preference of Actinobacteria for an alkaline pH (Atlas and Bartha 1997) was reflected by a significant decrease of their relative abundance with pH, from the Burren to the Wicklow soil (pH decrease from 6.45 to 4.45 in the order of Burren soil > Ardgillan soil > Wicklow soil). These observations agree with previous findings that, regardless of soil location, molecular studies seem to detect the same major phyla while the community structure at phylum level is highly site specific (Hackl et al. 2004).

Applying the phylotypic identities of baseline-ribotypes to their occurrence in fingerprints of the complete microcosm study (Liliensiek et al. 2012), treatment (i.e. plant species composition, fertilisation and presence of *Lolium perenne*; Additional file 2: Table S2) effects on the distribution of the baseline-communities were tested. Ribotypes defined as baseline communities in untreated bare soils generally were among the top 10, sometimes 20 most abundant ribotypes detected under any treatment condition within the same soil, their relative abundance distribution however was affected by treatment. Plant species was the main factor influencing most baseline ribo- and phyloypes, followed by fertilisation and the plant-fertilisation interaction (Table 2). The most pronounced plant species effect was observed in the Burren baseline-community with an influence on all ribotypes and phylotypes (F = 53.9 to 154.6 and F = 62.2 to 522. respectively), except one ribotype (F = 2.5). In the Ardgillan community 6 out of 10 ribotypes (F = 5.2 to 9.7) and only Actinobacteria (F = 4.1) displayed a significant response to plant species, while 5 out of 10 ribotypes (F = 3.9 to 32.7) and Acidobacteria and Firmicutes (F = 3.7 and 15.6, respectively) were influenced in the Wicklow soil. The *Lolium* effect was generally very weak, with only one ribotype influenced in the Burren and Ardgillan site (F = 4.7 and 3.0 respectively) while phylotypes seemed not to be affected. The Wicklow community showed no response to *Lolium* ingression, neither on the ribotype, nor phylotype level. A fertilisation effect on the phylotype level was only observed in the Burren and Wicklow soils, where it influenced Firmicutes (F = 12.3) only, and Firmicutes (F = 26.1) and Actinobacteria (F = 10.4), respectively. Similarly 5 and 6 ribotypes were influenced in these soils, respectively (F = 4.2 to 11.8 and 4.8 to 30.0, respectively), while only 1 ribotype was affected by fertilisation in the Ardgillan soil (F = 15.8).

**Table 2:** **ANOVA table of F-values for ribotype and phylotype responses to plant species, fertilisation and** ***Lolium perenne*** **ingression as well as their interactive effects**

	Ribotype	Phylotype	Plant	Fert	Plant x Fert	Lp	Plant x Lp	Fert x Lp	Plant x Fert x Lp
**Burren**	**225**	ni	2.5	1.8	1.3	0.0	4.2^**^	0.9	1.7
	**282**	An, αP, γP	53.9^***^	1.1	0.7	0.0	1.0	2.1	2.3
	**324**	ni	181.3^***^	0.3	3.9^**^	0.4	2.4	0.6	0.1
	**417**	An	88.7^***^	0.9	1.5	0.7	2.1	4.5^*^	4.3^**^
	**502**	γP	107.2^***^	5.5^*^	1.0	0.3	1.5	0.2	0.5
	**612**	ni	118.4^***^	11.7^**^	3.2^*^	4.7^*^	1.1	0.2	0.4
	**850**	F	140.2^***^	11.8^**^	5.5^**^	1.0	1.0	4.2^*^	1.2
	**853**	F	129.6^***^	11.0^**^	6.8^***^	0.1	3.2^*^	0.0	1.1
	**890**	ni	154.6^***^	0.7	0.9	1.0	1.7	0.2	0.5
	**895**	F	134.3^***^	4.2^*^	4.3^**^	1.8	7.1^***^	0.9	3.0^*^
	**Actinobacteria**		133.0^***^	0.1	1.4	0.1	2.3	0.0	0.5
	**Firmicutes**		522.0^***^	12.3^**^	9.3^***^	0.1	3.0^*^	0.4	0.5
	**α-Proteobacteria**		62.2^***^	1.0	0.8	0.0	1.1	1.9	2.2
	**γ-Proteobacteria**		92.6^***^	2.5	0.6	0.0	1.4	1.9	2.1
**Ardgillan**	**225**	ni	6.3^***^	2.1	7.7^***^	0.0	0.8	0.3	2.2
	**301**	An, F	8.4^***^	0.3	6.2^***^	0.6	5.6^**^	5.7^*^	6.9^***^
	**302**	An, F	1.7	0.5	0.3	0.5	0.9	0.8	0.6
	**400**	An	5.2^**^	0.0	2.1	0.6	0.1	0.4	1.0
	**831**	F	8.7^***^	0.4	0.7	3.1	0.7	2.9	0.6
	**835**	F	8.6^***^	0.1	0.6	1.3	0.7	3.4	0.2
	**843**	F	2.1	0.2	1.0	1.5	1.2	0.3	0.7
	**846**	ni	9.7^***^	15.8^***^	3.0^*^	7.6^**^	1.7	0.8	0.3
	**890**	ni	2.5	2.2	0.4	0.1	1.1	0.3	0.3
	**904**	F	1.4	0.4	0.9	0.2	2.0	0.1	0.5
	**Actinobacteria**		4.1^**^	0.9	6.5^***^	0.2	1.3	1.1	2.8^*^
	**Firmicutes**		1.3	0.6	4.2^**^	0.0	1.0	2.5	0.6
**Wicklow**	**225**	ni	13.8^***^	6.9^*^	9.8^***^	0.3	1.1	3.1	3.9^**^
	**241**	ni	3.9^**^	7.1^*^	1.6	1.2	1.8	0.0	1.0
	**352**	ni	32.7^***^	30.0^***^	9.9^***^	0.1	1.6	0.1	2.4
	**390**	An, F	10.8^***^	12.9^***^	1.9	0.9	1.0	9.1^**^	3.8^*^
	**391**	An, F	2.4	4.8^*^	0.5	0.6	1.0	0.0	1.6
	**398**	An, F	0.5	4.1	0.5	0.1	1.0	2.3	1.7
	**409**	An	2.3	0.3	0.4	0.1	1.0	0.0	1.3
	**917**	ni	12.0^***^	9.2^**^	7.1^***^	0.3	2.3	0.3	6.5^***^
	**920**	ni	1.9	2.4	2.0	1.0	2.3	2.6	0.4
	**953**	Ad	250227.0	52097.1	190238.3	17340.0	8134.4	49192.1	108320.4
	**Acidobacteria**		3.7^*^	0.8	2.9^*^	0.3	0.1	0.7	1.6
	**Actinobacteria**		2.4	10.4^**^	1.2	0.8	1.0	4.0	1.5
	**Firmicutes**		15.6^***^	26.1^***^	2.0	2.0	1.1	8.7^**^	2.3

Analysis of treatment effects on the baseline-communities of respective sites covered the full model of plant species (Plant), fertilisation (Fert) and Lolium ingression (Lp), including all their interactions (x). Ribo- and phylotypes belonging to the baseline-community of each site are listed in the first column, followed by F-values for each model term and their significance (p = 0.05 ≥ * > 0.01 ≥ ** > 0.001 ≥ ***).

*Abbreviations*: ni – not identified, An – Actinobacteria, Ad – Acidobacteria, F – Firmicutes, αP – α-Proteobacteria, γP – γ-Proteobacteria.

In summary, the constitution of the baseline community on ribotype and phylogenetic level showed some unique but mostly common members of each site and the distribution of relative abundances on both levels was significantly affected by site and treatment. While Proteobacteria were found solely in the baseline-community of the near neutral pH Burren soil, Acidobacteria were only found in the acidic Wicklow soil. The baseline-ribotypes in the Wicklow soil were less responsive to plant species and more susceptible to fertilisation compared to the other sites, while plant species, including *Lolium*, seemed to have the strongest effect in the Burren soil. In general interactive effects were less strong and common, suggesting a greater response to individual treatments. Critical evaluation brings up several issues associated to such an approach. Obviously, the specificity of identification is low due to limited homologies for environmental ITS genes in the database as well as the target sequence of ARISA clones. More interestingly, communities were dominated by Actinobacteria and Firmicutes which include genera with the highest RNA-operon copy numbers, raising the question, if studies considering only abundant ribotypes might overlook actually abundant species of the community.

## Electronic supplementary material

Additional file 1: Table S1: Origins and basic soil physico-chemical properties of the three unimproved grassland sites in Ireland. (PDF 12 KB)

Additional file 2: Table S2: Experimental design of complete microcosm study (Liliensiek *et al*., 2012). (PDF 79 KB)

Additional file 3: **Method details.** (PDF 110 KB)

Additional file 4: Table S3: Identities of cloned bacterial ARISA fragments matching the top 10 abundant ribotypes of bare untreated soils. (PDF 44 KB)
