# Supplementary material for: Linking phylogeny to abundant ribotypes of community fingerprints: an exercise on the phylotypic responses to plant species, fertilisation and Lolium perenne ingression
Source: Springerplus. 2013 Oct 9;2:522. doi: 10.1186/2193-1801-2-522 (PMC3824697; doi:10.1186/2193-1801-2-522)
Supplement: Supplementary file 1 — Additional file 1: Table S1: Origins and basic soil physico-chemical properties of the three unimproved grassland sites in Ireland. (PDF 12 KB) [file 40064_2013_596_MOESM1_ESM.pdf]

Table S1 Origins and basic soil physico-chemical properties of the three unimproved grassland sites in Ireland

	**Burren**	**Ardgillan**	**Wicklow**
**Location**	Burren National Park, Co. Clare	Ardgillan Castle, Balbriggan, Co. Dublin	Longhill, Kilmacanogue, Co. Wicklow
**National Grid Reference**	R 330 204	O 219 612	O 218 124
**Dominant Plant Species**	*Anthoxanthum odoratum, Briza media, Festuca ovina*	*Anthoxanthum odoratum, Agrostis capillaris, Holcus lanatus*	*Anthoxanthum odoratum, Agrostis capillaris, Festuca ovina*
**Sand**	33.39 ^{b}	48.67 ^{a}	34.37 ^{b}
**Clay**	34.59 ^{a}	31.09 ^{a}	27.75 ^{a}
**Silt**	32.03 ^{a}	20.23 ^{a}	37.88 ^{a}
**Soil Type**	Clay loam	Sandy clay loam	Clay loam
**pH**	6.35±0.02 ^{b}	5.75±0.01 ^{a}	4.45±0.01 ^{a}
**% N**	0.66±0.01 ^{b}	0.29±0.01 ^{a}	0.63±0.01 ^{c}
**% C**	7.44±0.18 ^{b}	3.95±0.23 ^{a}	9.57±0.22 ^{c}
**C:N**	11.19±0.17 ^{b}	13.50±0.38 ^{a}	15.26±0.16 ^{c}

Values representing within-site averages are shown. Within each row, different superscripts indicate significant group differences at level $p<0.05$.
