# Supplementary material for: Linking phylogeny to abundant ribotypes of community fingerprints: an exercise on the phylotypic responses to plant species, fertilisation and Lolium perenne ingression
Source: Springerplus. 2013 Oct 9;2:522. doi: 10.1186/2193-1801-2-522 (PMC3824697; doi:10.1186/2193-1801-2-522)
Supplement: Supplementary file 2 — Additional file 2: Table S2: Experimental design of complete microcosm study (Liliensiek et al., 2012). (PDF 79 KB) [file 40064_2013_596_MOESM2_ESM.pdf]

Table S2 Experimental design of complete microcosm study (Liliensiek *et al*., 2012)

**Site**	**Burren, Ardgillan, Wicklow**																			
**Plant**	**bare**				**Ao^{BAW}**				**Ac^{AW}/Bm^{B}**				**Fo^{BW}/Hl^{A}**				**mix**			
**NPK**	+		-		+		-		+		-		+		-		+		-	
Lolium	+	-	+	-	+	-	+	-	+	-	+	-	+	-	+	-	+	-	+	-

Soils from each site ("Site", Table S1) were sown ("Plant") with monocultures and mixtures of the respective three most abundant grass species (Table S1), and bare untreated soil (no plant, fertilization or *Lolium*) served as a control. The respective plant species were *Anthoxanthum odoratum* (Ao) for all three soils, *Agrostis capillaris* (Ac) for Ardgillan (A) and Wicklow (W), *Festuca ovina* (Fo) for Burren (B) and Wicklow (W), *Briza media* (Bm) for Burren (B) and *Holcus lanatus* (Hl) for Ardgillan (A). Each sowing regime received fertilisation ("NPK", +) or not (-) and additional sowing of *Lolium perenne* ("Lolium", +) or not (-). Each combination of treatments was replicated three times, giving a total of 180 pots. Pots were kept in a glasshouse for 75 days before sampling.

ARISA was carried out for each of the microcosms and bacterial ITS sequences of bare untreated soil (shaded cells) were randomly cloned and sequenced in this study. Phylogenetic identities as determined by cloning and sequencing, were matched to the 10 most abundant ribotypes of each bare untreated soil (Table S3) based on their sequence lengths, allowing a mismatch of ±5 bp on a weighted basis.
