# Supplementary material for: Linking phylogeny to abundant ribotypes of community fingerprints: an exercise on the phylotypic responses to plant species, fertilisation and Lolium perenne ingression
Source: Springerplus. 2013 Oct 9;2:522. doi: 10.1186/2193-1801-2-522 (PMC3824697; doi:10.1186/2193-1801-2-522)
Supplement: Supplementary file 3 — Additional file 3: Method details. (PDF 110 KB) [file 40064_2013_596_MOESM3_ESM.pdf]

Method details

Soil sampling for microcosms

Three unimproved grassland sites in Ireland were chosen for their different grassland and soil types. Site locations and their properties are summarized in Table S1. Soil samples were collected from the AH horizon (10-15 cm depth) of these sites in May and June 2007. Approximately 10 kg of soil were sampled from 20 random locations within each site, sieved to < 4 mm and used to set up microcosms within one week of sampling.

Microcosm set-up and sampling

The experimental design of the microcosm study is presented in Table S2. Microcosms were prepared by weighing 80 g (dry weight) of fresh soil into polyvinyl chloride pots (40 mm diameter, 110 mm height), which had been painted black to avoid penetration of light, and drilled at the bottom to allow free drainage of water. Soils of the three sites were sown with their respective three most abundant grass species (Emorsgate Seeds, Kings Lynn, United Kingdom). These were *Anthoxanthum odoratum*, *Agrostis capillaris*, and *Holcus lanatus* for the Ardgillan soil, *Anthoxanthum odoratum*, *Briza media* and *Festuca ovina* for the Burren soil and *Anthoxanthum odoratum*, *Agrostis capillaris*, and *Festuca ovina* for the Wicklow soil. Sowing regimes were such that each soil type was represented by 60 pots, of which twelve pots were bare soil, 12 monocultures of each of the respective species and 12 mixed cultures of all three respective species. Three pots of each of the sowing regimes were left untreated, three others were additionally sown with *Lolium perenne*, another three received NPK (10:10:20, 50 kg ha^{-1}) fertilisation at day 20, 40 and 60 and the last three received both, the additional sowing of *Lolium perenne* and NPK fertilisation. Microcosms were maintained in a greenhouse in a randomised block design for 75 days between June and August 2007. Water content was maintained at field moisture level of 30 % (w/w) by regular addition of distilled water. Plants were left untrimmed throughout the experiment and unsown plant

species were removed. Microcosms were destructively sampled 75 days after sowing. The roots were separated from the soil by sieving to < 4 mm.

DNA extraction

DNA was extracted based on a modification of the method by Griffiths *et al*. (2000). 0.5 g of sieved soil was added to 2 ml screw-cap tubes containing 0.5 g each of 0.1 mm silicon and 0.5 mm zirconia beads (Thistle Scientific Ltd., UK). 0.5 ml modified CTAB (hexadecyltrimethylammonium bromide, Sigma) extraction buffer (equal volumes of 10 % CTAB in 0.7 M NaCl with 240 mM K_2HPO_4, pH 8.0) was added followed by incubation at 70°C for 10 min in a water bath. After incubation, 0.5 ml phenol:chloroform:isoamylalcohol (Sigma, 25:24:1 (v/v)) was added and tubes were shaken in a ribolyser (Hybaid) at 5.5 m s^{-1} for 30 s. Following bead-beating, tubes were centrifuged at 16000 g for 5 min, after which the aqueous layer was transferred into a fresh set of tubes and residual phenol was extracted by mixing with equal volumes of chloroform:isoamylalcohol (Sigma, 24:1 (v/v)) twice. DNA was precipitated by the addition of 95 % ice cold ethanol and incubation at -20°C over night. Pellets resulting after centrifugation at 16000 g for 30 min at 4°C were washed with 70 % ethanol twice before resuspension in Tris-EDTA (pH 8). DNA extracts were visualised on a 0.8 % (w/v) agarose gel containing ethidium bromide (0.4 µg ml^{-1}) and quantified using a Nanodrop ND-1000 spectrophotometer (Thermo Scientific). All DNA extracts were diluted to yield 20 ng µl^{-1}.

Cloning

The 16S-23S intergenic space region from the bacterial rRNA operon was amplified using the unlabelled primer set ITSF/ ITSReub (Cardinale *et al.,* 2004). PCR reactions were performed in 25 µl volumes containing 20 ng DNA template. Reactions contained Go-Taq PCR buffer containing $MgCl_2$ (Promega, 1X, 1.5 mM $MgCl_2$), bovine serum albumin (New England Biolabs, 0.4 mg ml^{-1}), dNTPs (Promega, 200 µM), 15 pmol of each primer of the respective set (Invitrogen) and 1 U Go-Taq polymerase (Promega). Cycling conditions for the

ITSF/ITSReub primer set on a Px2 thermal cycler (Thermo Scientific) were 94°C for 3 min, 35 cycles of 94°C for 1 min, 61°C for 30 s, 72°C for 1 min and a final cycle of 72°C for 7 min. Products were quantified using a Nanodrop ND-1000 spectrophotometer (Thermo Scientific) and ligated into the vector pGEM-T Easy (Promega). The ligation reaction (12 µl) contained pGEM-T Easy vector (Promega, 50 ng), T4 ligase (Promega, 3 U), ligation buffer (Promega, 1X) and undiluted PCR products (4 µl) and was incubated overnight at 4°C.
Ligation products were then used to transform *E.coli* XL 10-Gold ultracompetent cells (U.S. Patent Nos. 5,512,468) by heat-shock. For the transformation 5 µl of ligation product were added to 100 µl of competent cells, which were then kept on ice for 30 min. Cells were then heat shocked at 42°C for 90 s and transferred onto ice immediately for further 2 min. Transformed cells were transferred to 800 µl Super Optimal Broth (SOB) (Bacto Tryptone 20 g l^{-1} Bacto Yeast 5 g l^{-1} NaCl 0.5 g l^{-1} KCl 0.186 g l^{-1}, pH 7.0) with catabolite repression (SOC) (970 ml l^{-1} SOB, $MgCl_2$ (20 mM), glucose (20 mM)), preheated to 37°C, and incubated at 37°C and 400 rpm for 1 hour.
Cultures were spread on sterile Luria Bertani (LB) (Bacto tryptone (10 g l^{-1}) Bacto Yeast (5 g l^{-1}) NaCl (5 g l^{-1})) agar (1.5 %) plates containing ampicillin (100 µg l^{-1}), IPTG (0.1 mM) and X-Gal (32 mg l^{-1}) and incubated at 37°C over night and 4°C for further 2 h before screening. White colonies were picked with sterile toothpicks and re-inoculated on fresh LB screening plates to exclude the possibility of false-positive results. Plates were incubated at 37°C overnight followed by 2 h at 4°C before picking confirmed white colonies for inoculation into LB broth containing ampicillin (100 µg l^{-1}). Cultures were grown at 37°C overnight.

Parallel to the LB cultures colony material was amplified by PCR using primers ITSF and ITSReub. Products were visualised on 2 % agarose gels containing ethidium bromide (0.4 µg ml^{-1}) to screen for products of the desired size. Product sizes were estimated using ImageMaster 1D Elite v.4.10 software (Amersham Pharmacia Biotech, Sweden).

Plasmids were extracted from cultures using the mi-Plasmid Mini Prep Kit (*Meta*Bion, Germany) according to manufacturers instructions. Extracts were quantified using a Nanodrop ND-1000 spectrophotometer (Thermo Scientific) and 1 µg of extract was dried for sequencing.

Sequencing

Plasmid inserts were sequenced bi-directionally using the primers T7 and SP6 (Macrogen Inc., Seoul, South Korea). Sequencing results for each clone were checked for consensus between both directions by reverse complementing one direction using the Sequence Utilities in BMC Search Launcher (http://searchlauncher.bcm.tmc.edu/seq-util/seq-util.html) and aligning both sequences using Multalign (http://bioinfo.genotoul.fr/multalin/multalin.html (Corpet 1988)). Any mismatches were checked by comparison of chromatograms, before submitting the 16S-ITS-23S consensus sequence (excluding primer binding sites) to the nucleotide database in BLAST (http://www.ncbi.nlm.nih.gov/blast/Blast.cgi (Altschul et al. 1997)). Tentative phyla/genera were assigned based on 16S-ITS-23S sequence matches with organisms contained in the database.

Matching sequences to ribotypes

Sequences derived by cloning and ribotypes were matched based on their exact matche of sequence length. Given a certain degree of inaccuracy during fragment analysis as well as sequencing it was decided to tolerate a length mismatch of ± 5 bp. In order to limit the resulting bias as much as possible matches between ribotypes and their respective clones were assigned on a weighted basis. This was done by multiplying the relative abundance of ribotypes exactly matching the sequence length of an identified sequence by 6, while relative abundances in a one bp mismatch were multiplied by 5 and in a 5 bp mismatch finally by one only. Resulting relative abundances were then averaged across the respective phyla to give an estimation of the phylum distribution.

Statistical Data Analysis

To give a comparison between sites/soil types, the experiment was analysed including samples from all three sites. To provide a more detailed view of the treatment effects, separate analyses were conducted within individual sites. Univariate analysis of variance (ANOVA) of biochemical data and diversity measurements was performed using the GLM procedure in SAS version 9.1. Group differences were determined using the Ryan–Einot–Gabriel–Welsch Multiple Range Test at significance level alpha <0.05, and pairwise comparisons were made using the estimate statement.

References

Altschul, S. F., T. L. Madden, A. A. Schaffer, J. Zhang, Z. Zhang, W. Miller and D. J. Lipman (1997). Gapped Blast and Psi-Blast: A New Generation of Protein Database Search Programs. Nucleic Acids Research **25**: 3389-3402.

Cardinale, M., L. Brusetti, P. Quatrini, S. Borin, A. M. Puglia, A. Rizzi, E. Zanardini, C. Sorlini, C. Corselli and D. Daffonchio (2004). Comparison of Different Primer Sets for Use in Automated Ribosomal Intergenic Spacer Analysis of Complex Bacterial Communities. Applied and Environmental Microbiology **70**(10): 6147-6156.

Corpet, F. (1988). Multiple Sequence Alignment with Hierarchical Clustering. Nucleic Acids Research **16**(22): 10881-10890.

Griffiths, R. I., A. S. Whiteley, A. G. O'Donnell and M. J. Bailey (2000). Rapid Method for Coextraction of DNA and RNA from Natural Environments for Analysis of Ribosomal DNA- and rRNA-Based Microbial Community Composition. Applied and Environmental Microbiology **66**(12): 5488-5491.
