# Supplementary material for: Linking phylogeny to abundant ribotypes of community fingerprints: an exercise on the phylotypic responses to plant species, fertilisation and Lolium perenne ingression
Source: Springerplus. 2013 Oct 9;2:522. doi: 10.1186/2193-1801-2-522 (PMC3824697; doi:10.1186/2193-1801-2-522)
Supplement: Supplementary file 4 — Additional file 4: Table S3: Identities of cloned bacterial ARISA fragments matching the top 10 abundant ribotypes of bare untreated soils. (PDF 44 KB) [file 40064_2013_596_MOESM4_ESM.pdf]

Table S3 Identities of cloned bacterial ARISA fragments matching the top 10 abundant ribotypes of bare untreated soils

Accession no.	sequence length	Matching ribotype(s)	Miss-match (+/-)	Closest NCBI match	Accession no.	Homology (%)	Overlap (nt)	Putative phylogenetic group
JQ229489	280	282	2	uncultured actinomycete strain CIU350-10	AJ417001	77	257	Actinobacteria
JQ229488	280	282	2	Uncultured actinobacterium clone LZWS1-05	DQ093151	89	240	Actinobacteria
JQ229473	281	282	1	Uncultured actinobacterium clone LZWS1-05	DQ093151	90	240	Actinobacteria
JQ229543	281	282	1	*Halomonas* sp. CA2-2I	AB367199	84	252	γ-Proteobacteria
JQ229482	282	282	0	*Sphingomonas wittichii* strain MA03	EU334830	89	55	α-Proteobacteria
JQ229475	283	282	1	Uncultured actinobacterium clone LZWS1-05	DQ093151	82	250	Actinobacteria
JQ229529	297	301, 302	4, 5	uncultured actinomycete strain CIU350-4	AJ417005	89	260	Actinobacteria
JQ229516	303	301, 302	2, 1	*Bacillus simplex* clone Bsim-1	AF478071	87	180	Firmicutes
JQ229502	387	390, 391	3, 4	uncultured actinomycete strain CIU350-10	AJ417003	82	154	Actinobacteria
JQ229497	393	390, 391, 398	3, 2, 5	*Heliobacterium modesticaldum* Ice1	CP000930	79	174	Firmicutes
JQ229526	401	398, 400	3, 1	*Frankia* sp. CWVEL	AF036902	90	65	Actinobacteria
JQ229520	402	398, 400	4, 2	*Frankia* sp. CWVEL	AF036901	88	75	Actinobacteria
JQ229534	413	398, 400	4, 2	Uncultured *Frankia* sp. isolate H1S2Hp	DQ988981	88	75	Actinobacteria
JQ229521	414	409, 417	4, 4	*Mycobacterium savoniae*	AJ748836	83	294	Actinobacteria
JQ229490	417	409, 417	5, 3	*Streptomyces scabiei* strain:DNK-G01	AB026214	78	137	Actinobacteria
JQ229540	420	409, 417	5, 3	*Streptomyces viridifaciens*	AY956512	95	49	Actinobacteria
JQ229484	506	502	4	*Xanthomonas campestris* pv. *vesicatoria*	AM039952	82	278	γ-Proteobacteria
JQ229492	834	831, 835	3, 1	*Alicyclobacillus acidocaldarius* subsp. *rittmannii* strain DSM 11297	EU723607	100	38	Firmicutes
JQ229479	838	835, 843	3, 5	*Alicyclobacillus acidocaldarius* subsp. *rittmannii* strain DSM 11297	EU723607	100	38	Firmicutes
JQ229512	853	850, 853	3, 0	*Alicyclobacillus acidocaldarius* subsp. *rittmannii* strain DSM 11297	EU723607	100	38	Firmicutes
JQ229474	856	853	3	*Alicyclobacillus acidocaldarius* subsp. *rittmannii* strain DSM 11297	EU723607	100	38	Firmicutes
JQ229486	900	895, 904	5, 4	*Alicyclobacillus acidocaldarius* subsp. *rittmannii* strain DSM 11297	EU723607	100	38	Firmicutes
JQ229533	954	953	1	Acidobacteria bacterium Ellin345	CP000360	77	388	Acidobacteria

Identities were acquired by random cloning of PCR products derived from PCR amplification using primers ITSF/ITSReub (Cardinale *et al.*, 2004), followed by bi-directional sequencing using primers SP6/T7 (Macrogen Inc.). Returned sequences were compared to sequences of known organisms using the nucleotide database on BLAST (Altschul *et al.*, 1997) and matched to the top 10 ribotypes of the three bare untreated soils. Out of the 27 ribotypes belonging to the top 10 of the three soils, 18 could be matched allowing a mismatch of +-5 bp. Ribotypes 225, 241, 352, 612, 846, 890, 917 and 920 did either not match any clones in length, or the matching clones did not produce any phylogenetic match in BLAST.
